# Valley Vortex Assisted and Topological Protected Microparticles Manipulation with Complicated 2D Patterns in a Star-like Sonic Crystal

**DOI:** 10.3390/ma14174939

**Published:** 2021-08-30

**Authors:** Jian Zhu, Tianning Chen, Chen Chen, Wei Ding

**Affiliations:** School of Mechanical Engineering, Xi’an Jiaotong University, Xi’an 710049, China; tnchen@mail.xjtu.edu.cn (T.C.); chen2017@stu.xjtu.edu.cn (C.C.); ddj3934@stu.xjtu.edu.cn (W.D.)

**Keywords:** metamaterials, topology, particle control, complicated pattern

## Abstract

Arranging microparticles into desired patterns, especially in a complicated pattern with a reliable and tunable manner, is challenging but highly desirable in the fields such as biomedicine and tissue engineering. To overcome these limitations, here, by using the concept of topology in acoustics, the valley vortex is utilized to manipulate particles on a large scale with complicated 2D patterns in the star-like sonic crystals at different frequencies. A topologically protected edge state is obtained at the interface of the crystals with different valley Hall phases, which shows the ability of reliable microparticles control along the sharp corner and the capability of robust particles cluster aggregation in a defective system. The results may provide intriguing resources for future microfluidic systems in a complicated and brittle environment.

## 1. Introduction

Precise, robust, and tunable microparticle assembly and manipulation has attracted much attention and has potential applications in numerous fields, such as biomedicine, bioanalytical chemistry, and tissue engineering [1,2,3,4,5,6]. The common optical tweezers, magnetic and electrostatic methods, etc., have successfully been used to manipulate particles, while the limitations of the weak force, high-power laser, biological sample damaging, etc., restrict their use in many applications [7,8,9]. An acoustic method offers an effective way to control the microparticles by using the interaction of sound with the particles and fluid inside the fluid due to its lab-free and biocompatibility [10,11,12]. In the field of acoustics, the most representative particle manipulation technique is the standing wave, represented by the two lab-on-chip methods, namely bulk acoustics wave (BAW) and surface acoustic wave (SAW) according to the actuation acoustic transducer. The BAW is usually generated by the piezoelectric transducer, and the SAW can be generated by the interdigitated transducer (IDTS) [6,10,11]. Many 1D (one-dimensional) alignments and 2D (two-dimensional) matrix particle patterns are designed effectively and rapidly by using one pair or two pairs of these acoustic transducers [13,14,15]. 

However, microparticle manipulation with the complicated patterns using the acoustic-driven standing wave is difficult due to its inherent and unavoidable limitations, such as the design and fabrication [6]. The design and fabrication of the acoustofluidic transducer such as the ITDS in SAW devices are complicated and expensive, especially in high-frequency regions [16,17]. Further, dedicated devices, such as the high-frequency power amplifier and the signal generator, are necessary. More importantly, the higher the frequency of the acoustic wave, the higher the particle resolution. However, the wave attenuation is very fast, and the propagation distance is limited, which results in limited experimental test regions and single or small area particle manipulation. 

Many other versatile acoustic wave-based methods are proposed for complicated shape particle design. For example, the monolithic acoustic holograms method is used to reconstruct the complex acoustic pressure fields to assemble the particles into asymmetric 2D shapes [18], the Chladni pattern formed over vibrating plates at different modes of the resonance, the hollow-shape microparticles patterning by the multimode of artificial materials [19,20,21,22], and the recent acoustic valley vortex state is also used to control and separate microparticles but at a fixed frequency and pattern [23]. The valley vortex states-based topological edge state has attracted much attention in condensed matter physics due to the backscattering-immunity of sound transport [24,25,26], which has been widely used to design various acoustic wave-based devices, such as the acoustic delay line and the robust programmable waveguide [27,28,29,30,31,32,33]. Very recently, the edge state formed at the interface of water and the 1D arrays of Helmholtz resonant air cavities showed that it can be used to control the particle by using the oscillations [34]. 

To overcome the limitations, e.g., limited acoustic field patterns and small operation regions of the traditional acoustic wave-based microparticles manipulation or the vortex-assisted sonic crystal at a fixed frequency, in this work, a kind of star-like sonic crystal is designed to control the particles with complicated patterns at different frequencies. Here, by using the concept of valley vortex states and protected edge states, we demonstrate that excited valley vortex states can be utilized to manipulate or assemble the particles on a large scale with complicated 2D patterns in the star-like sonic crystal at different states. In addition, a topologically protected edge state is obtained at the interface of the two types of star-like crystals, which shows the ability of reliable microparticles manipulation at the surface along the sharp corner. 

## 2. Structure and Design Methods

**Figure 1:**
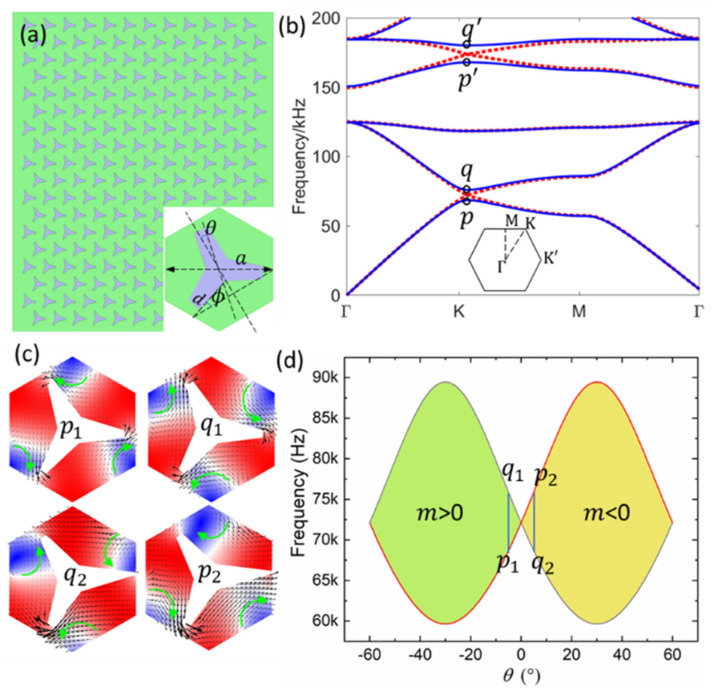
The configuration of the “star-like” sonic crystal and its vortex profile. (**a**) The sonic crystal consisted of a star-like structure embedded in the water. The enlarged unit cell of the crystal is shown in company. (**b**) The band structure for the case of θ=0° (red dotted line) and θ=5° (blue solid line) and the first Brillouin zone. (**c**) Calculated sound fields when θ=−5° and θ=5° at p, q states. The black and green arrows represent the energy and vortex profiles. (**d**) The band-edge frequencies varied with the rotation angle θ.

Figure 1a shows the configuration of the 2D sonic crystal (SC). The green area represents water in our case to simulate the environment of the acoustofluidic field. The SC consisted of a hexagonal-lattice array with a three-legged scatterer in the center. The enlargement in Figure 1a on the bottom right corner shows the unit cell of the crystal. The geometry had design parameters of a lattice constant a=43 mm, rotate angle θ=0/−5°, ϕ=18°, span d=0.6 mm. 

Figure 1b shows the calculated band structure by using the finite element method. The red dot lines represent the dispersion curve for the case of θ=0° where the crystal had the $C_{3v}$ symmetry, and the two Dirac points at the symmetry corner point K could be obtained simultaneously by controlling the scatterer and its geometrical parameters. By rotating the scatterer, for example, as indicated by the blue solid lines for the case of θ=5°, the $C_{3v}$ symmetry of the system reduced to $C_{3}$. When the mirror symmetry was broken it resulted in the degeneracy lift as indicated by the degenerated states p,q, and $p^{\prime },q^{\prime }$. Figure 1c shows the calculated sound field distributions at p, q states for the case of rotate θ=−5° (up parallel) and θ=5° (bottom parallel). The black arrows represent the acoustic energy flow which shows the vortex features. The vortex had an opposite chirality, i.e., the clockwise when θ=5° and anticlockwise when θ=−5°. The vortex chirality feature also can be observed in Figure 1d, which shows the band-edge frequencies varying with the rotation angle θ during the close and the reopening process of the bandgap. Furthermore, the different acoustic topological phases transition occurred, which can be characterized by the effective mass $m=sgn\left(\omega_{q1}-\omega_{p1}\right)$ [23,28,30]. 

## 3. Results

The valley states can be excited by the external sound, and the vortex in the acoustic system can offer a new way to manipulate the transport of sound or control microparticles. In this section, the vortex features, acoustic pressure field, and the acoustic radiation force are then numerically analyzed in the 2D sonic crystal system. We demonstrate the complicated 2D patterns that can be used to manipulate particles on a large scale at different frequencies. 

### 3.1. 2D Patterns and Particles Aggregation in the Sonic Crystals at the Different States

**Figure 2:**
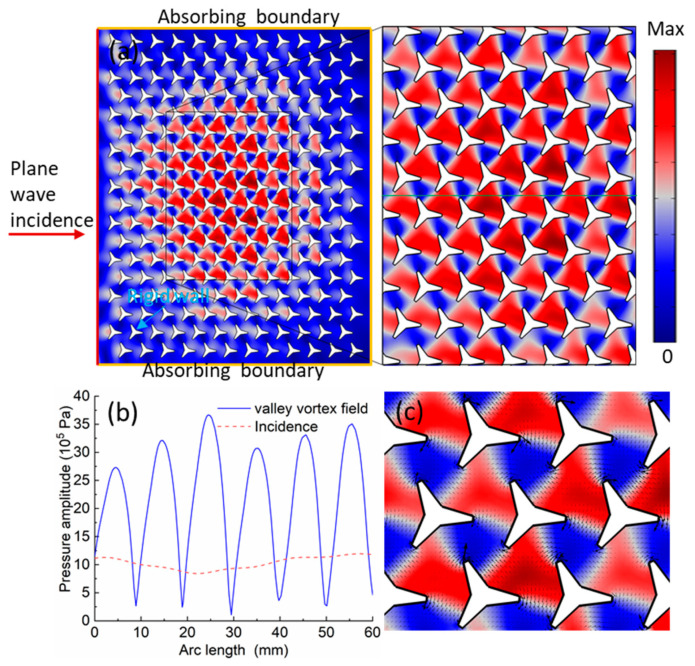
The acoustic field distribution and acoustic radiation force calculation. (**a**) The simulation model and the calculated acoustic pressure fields and its enlarged view when the plane wave was incident from left to right. (**b**) The extracted amplitude of the incidence (the red dash line) and the excited valley vortex field (the blue line) from the green line in (**a**). (**c**) The calculated acoustic radiation force potential and force.

The valley vortex state in the crystal can be generated by the external sound excitation, and the exciting incident angle can be determined according to the conservation of the momentum parallel to the crystal interface [24]. Figure 2 shows the calculated acoustic pressure field and acoustic radiation force. In the simulation model, a plane wave excitation was applied on the left boundary. The absorbing boundary conditions were set at the top, bottom, and right boundaries to absorb the acoustic energy, as shown in Figure 2a. Moreover, the star-like scatters were assumed to be rigid wall boundaries. The largest mesh size in the simulation was smaller than one-tenth of the shortest incident wavelength. Figure 2a shows the calculated acoustic pressure field distribution when the plane wave with a frequency of 66.9 kHz was incident from left to right. We noticed that the external sound excitation was slightly different from the theoretically predicted result in Figure 1b, with a maximum tolerance of 1.7% and 0.1% for *p* and *p*′ states, respectively. The reason why the theoretical predicted and simulated results are slightly different is due to the unavoidable uncertainty of the mismatch of the acoustic impedance [23,35] The distinct pressure nodes and antinodes were obtained, which can be used to control the trajectories and locations of the microparticles. The enlarged view of the field is shown in the company on the right. In Figure 2b, we extracted the acoustic pressure amplitudes from the green line in Figure 2a, the red dash line represents the incidence, and the blue solid line is the excited valley vortex field. The results showed that the amplitude of the excited pressure could be about 3.5 times as larger as the incidence due to the local resonance of the periodic sonic crystal structures. 

After obtaining the acoustic pressure fields, we further calculated the acoustic pressure-induced acoustic radiation force according to the theory of Gor’kov [11,36,37]. The acoustic radiation force acting on a particle, $F_{r}$, comes from the scattering of the ultrasound waves on the particle and is induced by radiation force potential field U, which can be expressed as [37,38]

(1) $$U\;=V_{0}\left[f_{1}\frac{1}{4\rho_{f}c_{f}^{2}}Re\left(p\cdot p^{*}\right)-f_{2}\frac{3\rho_{f}}{8}Re\left(v\cdot v^{*}\right)\right]$$

(2) $$F_{r}=-\nabla U$$

with factors $f_{1}=1-\frac{k_{p}}{k_{f}}$ and $f_{2}=2\left(\rho_{p}-\rho_{f}\right)/\left(2\rho_{p}+\rho_{f}\right)$. $V_{0}$ is the volume of the particle, $\rho_{f},$ and $k_{f}$ are the density and compressibility of the fluid, the subscripts f and p denote the fluid and particle, p and v are the calculated acoustic pressure and velocity fields. 

The above problems were solved by utilizing the finite element method. The Comsol Multiphysics software was used in this case to calculate the radiation force potential field and the acoustic radiation force. The results are shown in Figure 2c, in which the 2D patterns represent the radiation force potential field, and the black arrows represent the radiation force. The direction of the arrow shows that the radiation force direction exhibited from high to low (red to the blue area) force potential field, which is an acoustic radiation characteristic.

We can further mimic the particle distribution and locations after obtaining the acoustic radiation force in Figure 2. Here we also considered the viscous effects of the particle in the fluid. Only drag forces were considered in this work due to the calculated domain being much larger than the wavelength and the boundary layers. The drag force can be expressed as [37,38]

(3) $$\;F_{s}=\;-6\pi \eta R\left(v_{p}-v_{f}\right)$$

where η and R represent the viscosity of the fluid and the radius of the particle, $v_{p}$ and $v_{f}$ denote the velocity of the particle and the fluid, respectively. We assumed that the microparticles were suspended on the fluid and the surrounding background was static, and the gravity equaled to the footage. 

**Figure 3:**
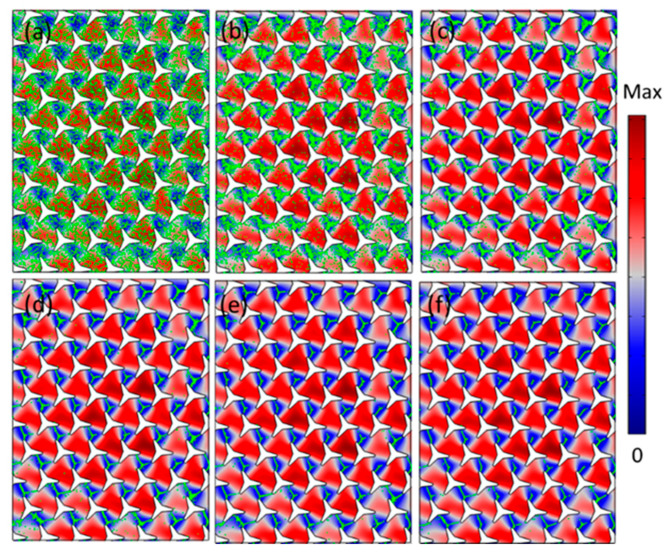
Numerical validation of the microparticles patterning and locations when the incident wave was excited at the frequency of 66.9 kHz. (**a**–**f**) The microparticles distribution at the stating positions when t=0 s, t=1 s, t=2 s, t=3 s, t=4 s, and t=5 s.

Figure 3 shows the microparticles distribution and locations along with times when a plane wave was incident from left to right. The background fluid was water with a density $\rho_{p}=1000\;\mathrm{kg}/m^{3}$, modulus K=2.25 GPa. The particle was selected as polystyrene, which is commonly used in the field of microfluidic and polymer with physical parameters $\rho_{p}=1050\;\mathrm{kg}/m^{3}$, pressure-wave speed $c_{p}=2350\;m/s$, a=20 um. Figure 3a represents 15,000 particles (green points) that were randomly distributed in the calculated area at the starting positions when t=0 s. The acoustic field pattern and radiation force were obtained based on Figure 2 with the incident frequency of 66.9 kHz. The particles were gradually moved to the acoustic valley when we turned on the acoustic waves, as shown in Figure 3b–f, in which the distribution and locations of the particles are demonstrated at t=1, t=2, t=3, t=4 and t=5 s. 

**Figure 4:**
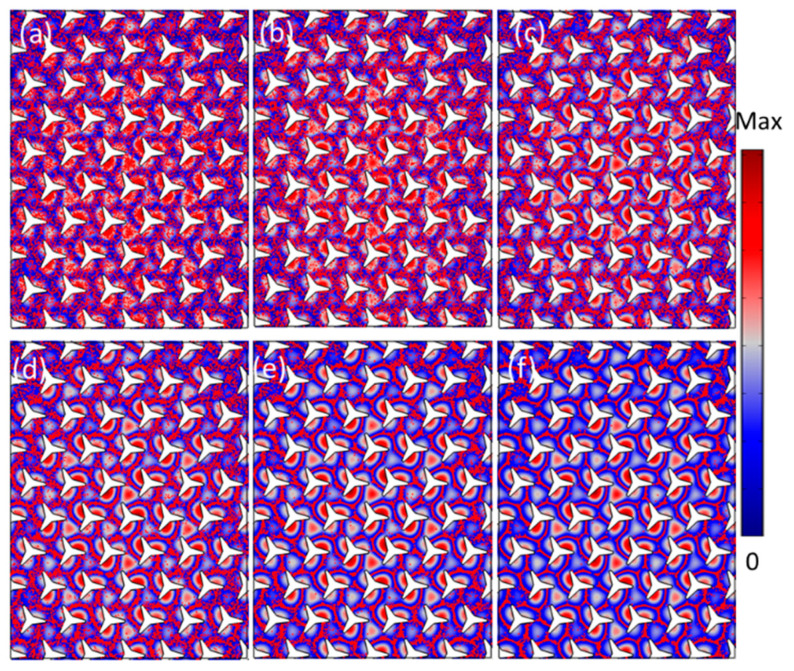
Numerical validation of the microparticles patterning and locations for the case of the incident wave at the frequency of 167.6 kHz. (**a**–**f**) The microparticles distribution at the stating positions when t=0 s, t=1 s t=2 s t=3 s t=5 s, and t=8 s.

Further, we calculated the location tracing of the particles when the incidence at another vortex/Dirac point with the frequency at 167.6 kHz, as shown in Figure 4. It is the particle distribution and locations when t=0, t=1, t=2, t=3, t=5, and t=8 s. Compared to the results in Figure 3, a more complicated star-like shape pattern was obtained. It cannot be easily obtained with traditional acoustofluidic methods, such as standing wave or traveling wave-based acoustic techniques.

### 3.2. Topologically Protected Edge State and Its Microparticles Control along the Sharp Corner

Next, we further explored the manipulation of microparticles by using the valley vortex states-based topological edge state, which owns the backscattering immunity of sound transport. The particle was selected as polydimethylsiloxane with physics parameters $\rho_{p}=965\;\mathrm{kg}/m^{3}$, pressure-wave speed $c_{p}=1080\;m/s$, a=20 μm.

**Figure 5:**
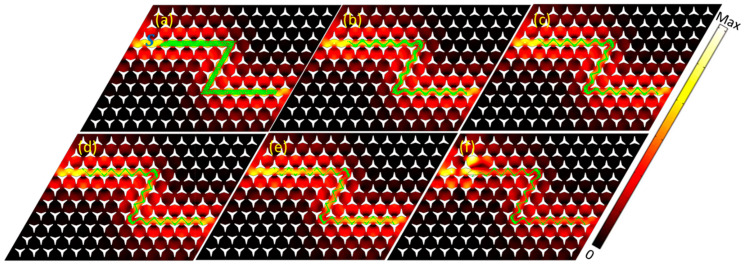
Microparticle distribution at the topologically protected edge states. (**a**) A point source was excited at S with the incident frequency of 66.9 kHz, the green points are the microparticles at initial locations when t=0 s. (**b**–**e**) For the case of when t=1 s, t=1.5 s, t=2 s, and t=3 s. (**f**) For the case of t=3 s with a defect at the interface of the structures.

Figure 5 shows the particle control at the edge states along the sharp corner (zigzag curve). The zigzag curve and edge state were formed at the interface of the two domains, where the two types of sonic crystals had rotation angles of θ=−30° and θ=30°, respectively. The excitation was a point source located at *S*, as shown in Figure 5a, in which the acoustic wave could propagate smoothly along the zigzag curve without any backscattering. The green points represent 5000 particles randomly distributed in the calculated area at the interface when t=0 s. Figure 5b shows the particle distribution and locations when we turned on the acoustic waves when t=1 s. The particles were gradually moving toward the nodes of the acoustic field pattern under the acoustic radiation force and drag force. Figure 5c–e shows the particle distribution varied with time when t=1.5 s, t=2 s, and t=3 s, respectively. An intriguing sawtooth-type particle patterning was obtained in the zigzag curve interface which was formed along the distribution of the acoustic pressure field in the channel. Figure 5e shows the robust particles cluster aggregation when t=3 s, even when the structures with a defect at the interface. 

## 4. Discussion and Conclusions

In summary, the sonic crystal (SC) is a kind of artificially periodic structure with the ability of wave or energy control in many fields, e.g., noise barrier [39,40], acoustic and vibration attenuation [41], which has attracted much attention. Here, in this work, we further constructed a type of star-like sonic crystal (SC) to show its capability of microparticle control in water application by using the concept of valley vortex states and topologically protected edge states in SC, which cannot be easily realized by using traditional acoustofluidic methods or the water-borne wave control by utilizing composite metamaterials [42,43]. The designed SC consisted of a hexagonal-lattice array with a three-legged scatterer in the center. The corresponding frequencies, bandwidth, vortex, etc., can be simply manipulated by scatterers operations. We demonstrated the excited valley vortex can be utilized to manipulate particles with complicated 2D patterns in the sonic crystals at different states. The complicated star-like shape pattern was obtained, which cannot be easily obtained with traditional acoustofluidic methods, such as standing wave or traveling wave-based acoustic devices. In addition, a topologically protected edge state was obtained at the interface of the two types of crystals, which shows the ability of reliable microparticles control along the sharp corner. Furthermore, it has the capability of robust particles cluster aggregation in a defective system. The results may promote the real applications of the acoustic topological insulators and provide an alternative way for particle manipulation in the future microfluidic system in a complicated and brittle environment.

## Data Availability

The data presented in this study are available on reasonable request from the corresponding author.
